# Influence of climate variation on phenolic composition and antioxidant capacity of *Medicago minima* populations

**DOI:** 10.1038/s41598-020-65160-4

**Published:** 2020-05-19

**Authors:** Souhir Kabtni, Dorra Sdouga, Ines Bettaib Rebey, Mattew Save, Neila Trifi-Farah, Marie-Laure Fauconnier, Sonia Marghali

**Affiliations:** 1 Laboratory of Molecular Genetics, Immunology & Biotechnology, Faculty of Sciences of Tunis, University of Tunis El Manar, Tunis, Tunisia; 2 General and Organic Chemistry-Volatolomics, Gembloux Agro-Bio Tech, University of Liège, Liège, Belgium

**Keywords:** Natural variation in plants, Environmental impact

## Abstract

*Medicago minima* is a pasture legume that grows almost all over the world. In Tunisia, it occupies various climatic environments and is considered the most abundant annual *Medicago* plant. However, this species is unconsumed and unused by humans. This study aimed to explore the phytochemical characteristics of *Medicago minima* selected from different provenances in Tunisia and subsequently investigate the influence of environmental factors on their phenolic composition and antioxidant activity. Therefore, a calorimetric method and DPPH tests provided the total phenolic and total flavonoid contents and antioxidant potential in roots, stems, leaves and seeds. High performance liquid chromatography (HPLC) identified and quantified four phenolic acids and three flavonoids in the studied organs. Roots and leaves showed the greatest phenolic compound content and had high antioxidant activity. Rutin and syringic acid (leaves) represent a characteristic for this species. For each organ, principal component analysis of phenolic profiles showed that the root’s phenolic composition could be an indication of the plant adaptation to even small changes in its environments. Plants originating from a cold climate, higher altitude or semi-arid environment had the highest phenolic compound contents in their organs. Our findings provide useful information for the exploitation of the phenolic compounds in these weeds for the development of environmental sustainability.

## Introduction

Fabaceae is an important taxonomic class that includes numerous species consumed by both humans and animals, such as clover, peanut, alfalfa and various beans. The *Medicago* genus belongs to the Leguminosae family and presents a wide range of interesting species for foods and feeds. These species are reported in many studies as sources of phytochemical compounds, including carotenoids, saponins, flavonoids and phytoestrogens^1–3^. These potential active ingredients have a significant preventive role against chronic diseases, especially when they are absorbed through the diet^4,5^. Otherwise, the biochemical potential of some *Medicago* species remains unknown. *Medicago minima* is a pasture species that grows in cultivated and uncultivated fields, mountain pastures almost all over the world, North Africa, Asia, Europe and the USA^6–9^. Although some studies have described this species as bioactive isoflavone sources^1,2^, the use of this species remains limited to pastoral animals. Therefore, *M. minima* is unconsumed and unused by humans. Furthermore, this species is widespread in semiarid temperate regions, especially in the Mediterranean Basin^6^. It tolerates the most varied edaphic and climatic conditions^6,8^. In Tunisia, *M. minima* resides in various climatic environments, namely semi-arid and Mediterranean climates, and they are the most abundant annual *Medicago* plants^10^. The adaptation of *M. minima* to its environments can impact its secondary metabolism. In addition, according to previous studies, plants manage climate change by the variation of their secondary metabolism^11,12^. This adaptive strategy is a complex mechanism of physiological and molecular programmes that involves several genes and pathways^11^. In response to drought, several plants increase their phenolic contents and decrease protein and carbon metabolites^13^. However, other plants increase their sugar and carbon metabolites and some phenolic acids^11,13^. Typically, plant cells synthesise a wide range of active ingredients, classified as primary and secondary metabolites, that are involved in their adaptation to the various environments^5,11^. In this study, we focused on secondary metabolism as active compounds.

Secondary metabolic compounds have a high chemical diversity, and phenolic compounds are one of the most varied groups^12^. Phenolic acids and flavonoids are widely distributed in nature and can be isolated from vegetables, herbs and fruits. Due to their therapeutic potential, phenolic compounds have received considerable attention in scientific research^14–18^. In fact, scientific experiments support their beneficial role in bodily health^14–18^. Recent studies have reported that many biological activities are linked to the presence of phenolic compounds, which act as free radical scavengers and/or metal chelators^15–17^. Some studies have reported that luteolin (flavone) induces apoptosis in cancer cells^19^, but it is already used mostly for treating cardiovascular diseases, relieving cough and eliminating phlegm^18^. Sinapic acid and syringic acid also have great potential to attenuate various chemically induced toxicities and mucosal carcinogenesis. These substances have been shown in many biological activities^20–22^. Natural compounds are usually described as non-toxic to humans and other organisms. However, it is necessary to note that the extensive uses of these active composites may have toxic effects^22^. For this purpose, many studies have been interested in identifying these natural active compounds in plants and then determine their contents and their spectrum of functional properties. Nonetheless, most studies have only focused only medicinal and aromatic plants^5,15,17,23^, whereas some species have so far remained unexplored. Furthermore, most of the studies have been interested in the biochemical composition of one or two plant parts^13,23–25^, a focus that can omit a piece of fascinating information about the studied species. To better determine the values of unknown *Medicago* species in scientific research and the development of medicinal resources, it is necessary to investigate their phenolic composition in all plant parts. Therefore, the first objective of this study was to highlight the distribution of phenolic compounds in various *M. minima* organs and establish their antioxidant capacity. Our objective was to determine the total phenolic and total flavonoid contents and identify several phenolic and flavonoid compounds in roots, stem, leaves and seeds of *M. minima* populations. The second purpose was to create a comprehensive connection between *M. minima* adaptations to various environmental factors (climate and altitude) and the levels of phenolic compounds in their organs. Therefore, we aimed to discriminate twelve *M. minima* populations selected from different Tunisian climatic environments according to their phenolic profiles.

## Results

### Total phenolic contents (TPC)

**Figure 1:**
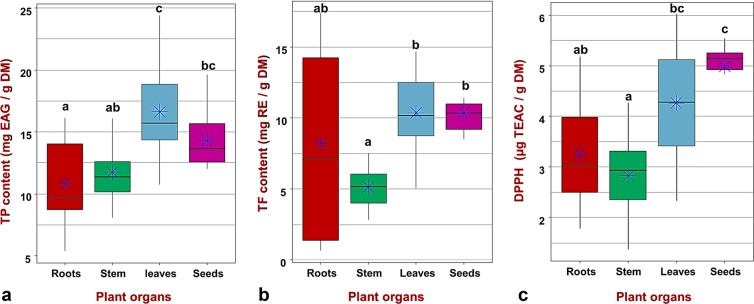
The distribution of the TPC and the TFC in four organs of the *Medicago minima* species and their DPPH radical scavenging activity. (**a**) The distribution of the total phenolic contents. (**b**) The distribution of the total flavonoids contents. (**c**) The antioxidant capacity of four plant organs. The blue asterisk indicate the means. Each data point was present with the average of twelve means ± SD. Labelled columns not connected by the same letter are significantly different at P < 0.05, based on a Tukey’s honestly significant difference test. Figures were carried out using R program packages (tidyverse and hrbrthemes).

**Figure 2:**
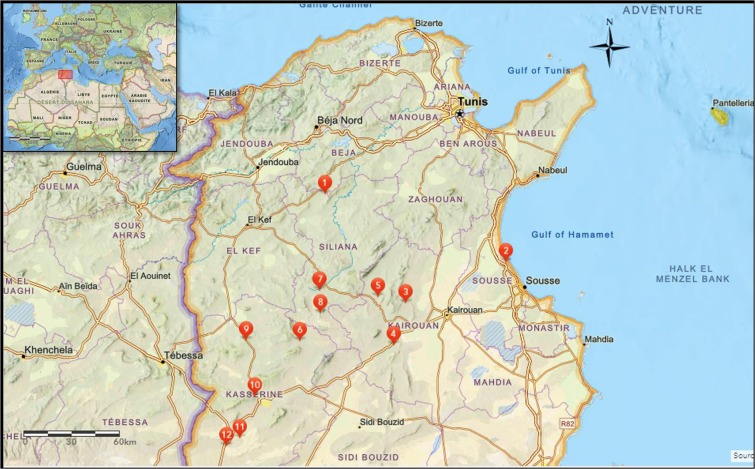
Geographic locations of the collection sites of Tunisian *Medicago minima* populations. Maps generated by ESRI, ArcGis Online tools. The numbers correspond to the collection sites and its details are summarised in Supplementary Table S3.

**Figure 3:**
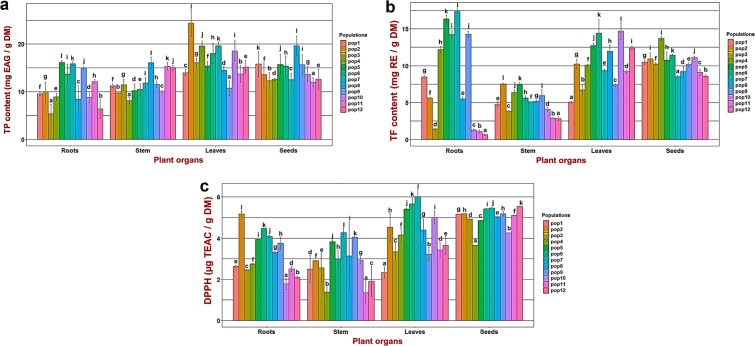
The variability of leaves, stems, roots, and seeds extracts among twelve *Medicago minima* populations. (**a**) The variability of the total phenolic contents in four organs among *Medicago minima* populations. We expressed the TP contents as milligrams of gallic acid equivalents per gram of dry weight. (**b**) The variability of the total flavonoid contents in four organs among *Medicago minima* populations. The TF contents is expressed as milligrams of rutin equivalents per gram of dry weight. (**c**) The variability of the antioxidant capacity (DPPH) in four organs among *Medicago minima* populations. The DPPH results are expressed as mg of Trolox equivalent antioxidant capacity (TEAC) per gram of dry weight. Each population is represented by a characteristic colour. Each data point was present with the average of triplicate experiments ± SD. In each organ (same colour), the population’s means were compared using the statistical method of the Honest Significant Difference (HSD) and labelled columns not connected by the same letter are significantly different at p < 0.05. Histograms were carried out with tidyverse and ggplot2 R program packages.

The TPC of *M. minima* roots, stems, leaves and seeds extracts were analysed and are displayed in Fig. 1. The statistical analysis indicated a significant difference between the TPC of studied organs (Supplementary Table S1). These results demonstrated that leaf and seed extracts have the highest TPC, respectively, with 16.65 mg GAE/g DM and 15.97 mg GAE/g DM (Fig. 1a), whereas both root and stem extracts have the lowest contents. Therefore, *M. minima* accumulates more phenolic compounds in their aerial organs, especially in their leaves and seeds. To identify which plant organs are different between studied populations (Fig. 2), we performed analysis of variance (ANOVA), coupled with the Tukey test, separately for each organ (Fig. 3, Supplementary Table S2). The TPC differed significantly between and within plants parts (p < 0.001). In addition, given the geographical closeness of Kairouan populations (pop5 and pop3), we contrasted their root TPC; we observed the same results in Siliana populations (pop7 and pop8) (Figs. 2, 3a, Supplementary Tables S3, S4). Similarly, despite the geographic closeness between pop10 and pop11, their TPC were dissimilar in the leaves and stems (Fig. 2, Supplementary Table S4). However, all populations exhibited higher TPC in their seed extracts compared to the root and stem extracts. In each organ, the variation of the TPC among studied populations was not associated with the geographic distance. Nevertheless, the populations with a considerable TPC are from a BSK climate (arid-cold-steppe) with a higher altitude (Supplementary Table S3, Fig. 3). These populations are located in the Kasserine region (pop6, pop9 and pop11), with an altitude range of 720–1009 m, Siliana (pop8) and Kairouan (pop5) (Fig. 3, Supplementary Table S3).

### Total flavonoid contents (TFC)

The analysed results of the TFC highlighted the presence of a significant difference among the various *M. minima* organs (Fig. 1). These results were statistically validated by the Kruskal-Wallis rank-sum test (p = 0.001907) and ANOVA (p = 0.00336) coupled with Tukey test (Fig. 1b, Supplementary Table S1). The leaf and seed extracts had the highest TFC, with 10.35 mg RE/g DM, whereas the stem presented a lower content. If we focus on each organ individually, there was a statistically significant difference among the TFC of various populations (Supplementary Table S2).

Indeed, pop9, pop5 and pop7 presented the highest root TFC. Their root TFC exceeded the values measured in their leaves and seeds (Figs. 2, 3a). In addition, pop2, pop5, pop6, and pop9 exhibited a high upper stem TFC (Fig. 3b). Those populations reside in the lower-semi-arid zone and, geographically, they are separated from populations with a medium TFC level from those with a lower TFC. These findings establish a continuous line on the map (Figs. 2, 3b). Therefore, populations from the lower semi-arid zone with a cold-steppe climate had the highest stems’ TFC, greater than 5 mg RE/g DM. In contrast, the populations with the lowest contents inhabit higher arid regions with a cold-steppe climate (Fig. 3, Supplementary Table S3). Similarly, pop10, pop6 and pop5 had the highest leaf TFC. For the seed extracts, the highest TFC was more than 11 mg RE/g DM; pop12 and pop6 presented the highest values (Fig. 3b).

In summary, all studied *M. minima* populations exhibited statistically significant differences in TFC. All of populations from Kasserine (pop6 and pop9) and Kairouan (pop4 and pop5) exhibited the highest TFC values. Therefore, adaptation to the lower semi-arid region with a cold-steppe climate seems to be associated with higher TFC.

### The identification and the quantification of some phenolic compounds by HPLC-DAD

**Figure 4:**
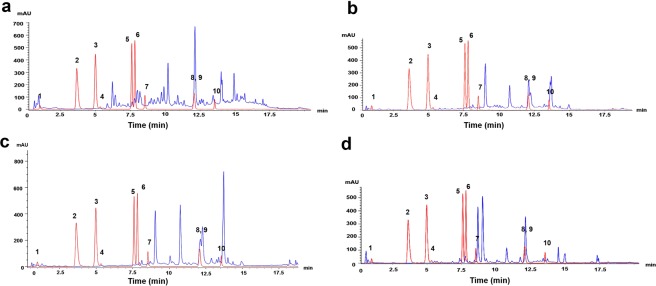
HPLC profiles of the identification of phenolic acids and flavonoids in different plant parts: detected in 315.4 nm wavelength. The blue chromatogram is the extract chromatogram profile with (**a**) the chromatogram of roots extract, (**b**) the chromatogram of stems extract, (**c**) the chromatogram of leaves extract and (**d**) the chromatogram of seeds extract. The red chromatogram is the standards chromatogram profile with **1:** gallic acid, **2:** gentisic acid, **3:** caffeic acid, **4:** syringic acid, **5:** ferulic acid, **6:** sinapic acid, **7:** rutin, **8,9:** quercetin, luteolin, **10:** kaempferol. The software Agilent ChemStation carried out the chromatograms.

**Figure 5:**
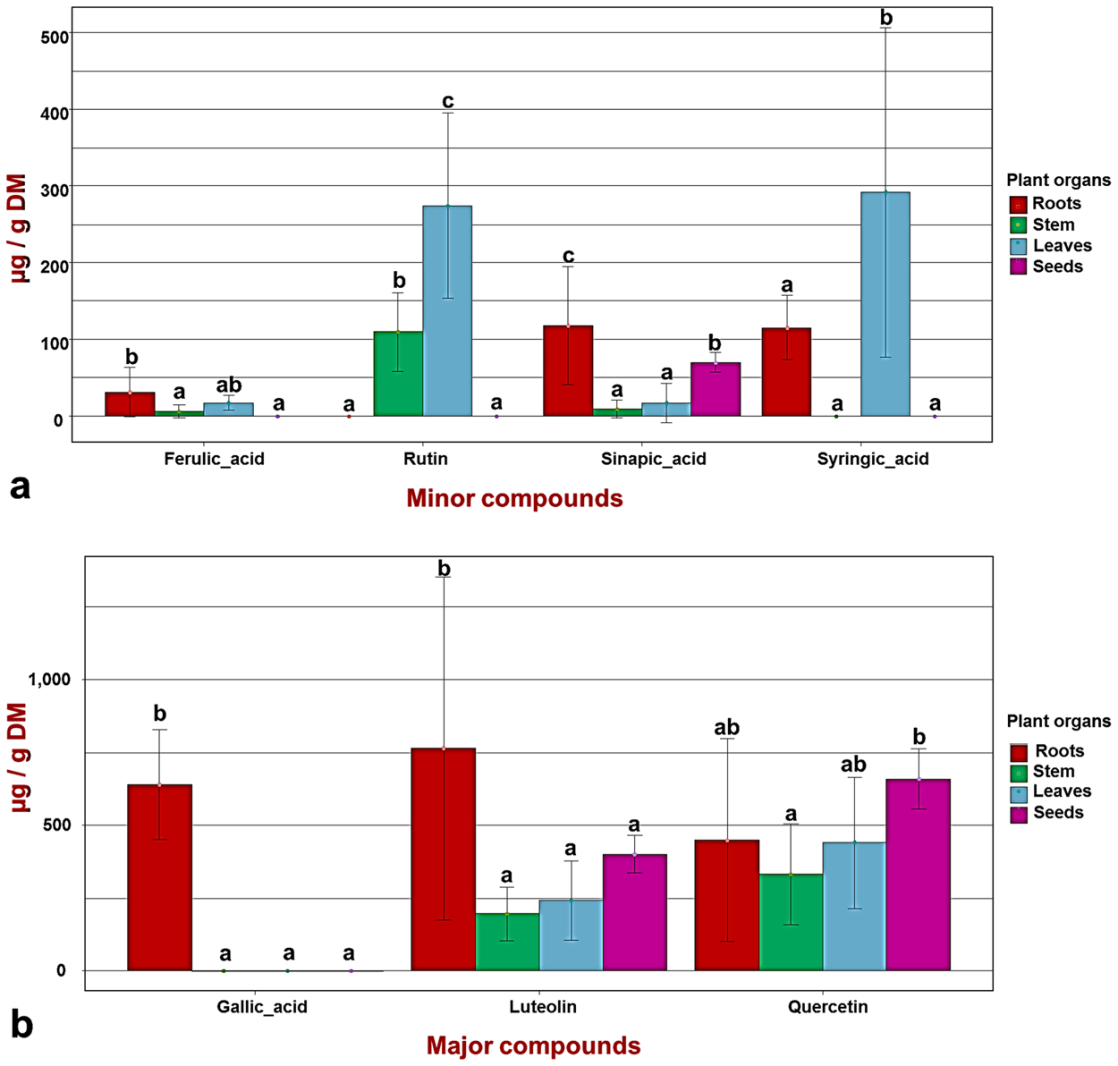
The variance of the phenolic compounds among *Medicago minima* organ extracts. (**a**) The minor compounds in *Medicago minima* plant organs. (**b**) The major compounds in *Medicago minima* plant organs. Each data point was present with the average of twelve means ± SD. For each compounds, labelled columns not connected by the same letter are significantly different at P < 0.05, based on a Tukey’s honestly significant difference test. The tidyverse and ggplot2 R program packages produced figures.

HPLC analyses of various *M. minima* extracts presented profiles with a retention time that coincide precisely with seven compounds (Fig. 4). Statistical analysis of the identified compounds contents revealed a wide range of differences between various plant organs (p < 0.05) (Supplementary Table S1). Figure 5 presents the marked variation in the phenolic compound contents among plant parts; it suggests the presence of major (Fig. 5b) and minor (Fig. 5a) compounds. In fact, the major identified compounds correspond to the gallic acid, luteoline and quercetin. Those compounds were present in the plants’ parts with an average concentration between 250 and 750 µg/g DM (Fig. 5b). The identified minor compounds included ferulic acid, rutin, sinapic acid and syringic acid, with an average concentration less than 300 µg/g DM (Fig. 5a). The variability among plant parts was highlighted by the presence of the gallic acid only in the root extract, rutin only in the stem and leaf extracts and syringic acid only in root and leaf extracts (Fig. 5). The lack of ferulic acid in the seeds extracts is also remarkable.

**Figure 6:**
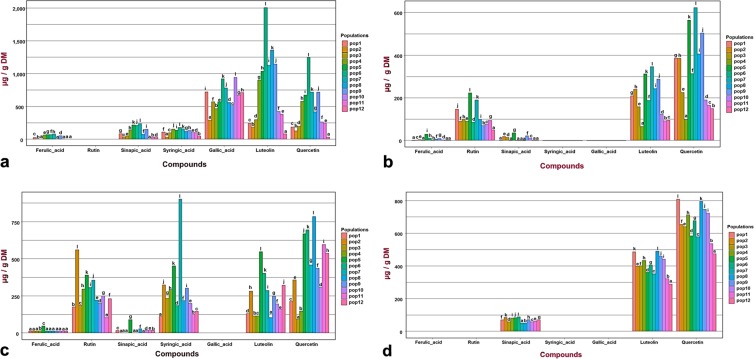
The phenolic compounds in each plant organ and their variance among populations. For each compound, the population’s contents were compared and, labelled columns not connected by the same letter are significantly different at P < 0.05, based on a Tukey’s honestly significant difference test. Figures were produced by the tidyverse and the ggplot2 R program packages.

Almost all of major and minor compounds were present in the *M. minima* roots, except rutin (Fig. 5). There was also variation in the contents of these compounds within the root extracts (Supplementary Table S2). Ferulic acid and sinapic acid were produced in very small quantities in the population roots from Sousse (pop2), Kairouan (pop3) and Kasserine (pop10, pop11 and pop12) (Figs. 2, 6). Those populations contained the amount of the identified compounds and inhabit higher arid zones with a cold-steppe climate or the lower semi-arid zone with a Mediterranean climate (Fig. 6, Supplementary Table S3). Most of the populations with high root extract contents were from pop5, pop7 and pop6, all of which represent the Tunisian dorsal mountain in the lower semi-arid region with a cold-steppe climate. In the stems, the presence of rutin and the absence of syringic acid and gallic acid were notable (Fig. 5). Statistical analysis highlighted a variation within the stem extracts (Supplementary Table S2). The populations from lower semi-arid regions with a cold-steppe climate (pop5, pop7, pop8 and pop9) exhibited the highest phenolic compound contents. In contrast, populations from the higher arid climate (pop4, pop11 and pop12) presented the lowest contents (Fig. 6, Supplementary Table S3). Like the stem extracts, the leaf extracts did not contain gallic acid, but they did exhibit a small amount of ferulic acid and sinapic acid (Fig. 6). In addition, the identified phenolic compounds were present in the leaf extracts, albeit at variable quantities. Hence, there was a significant difference between populations (p < 0.05) (Fig. 6, Supplementary Table S2). Pop5, pop6 and pop7 from the centre of the Tunisian dorsal mountain and distributed in the low-semi-arid region with higher altitude (higher than 550 m; Supplementary Table S3) presented the highest leaf contents (Fig. 6). For seeds, only sinapic acid, luteolin and quercetin were detected and quantified (Fig. 5). Quercetin and luteolin constituted the major compounds identified in the seed extracts (Fig. 6). Otherwise, in the lower semi-arid zone, the Tunisian dorsal mountain populations (pop8, pop9 and pop10) showed higher contents of those compounds.

Our results suggested that the roots and the leaves represent the most abundant contents of active compounds (Figs. 3, 4). Moreover, based on ANOVA, there was great variability (p < 0.001) in the contents among the studied populations (Supplementary Table S2). The populations with the highest contents of known compounds inhabit the Tunisian dorsal mountains in the lower-semi-arid region with a cold-steppe climate. In contrast, populations with lower contents are distributed in the higher-arid region of the Tunisian dorsal mountains with a cold-steppe climate.

### Antioxidant activity by free radical scavenging (DPPH)

**Figure 7:**
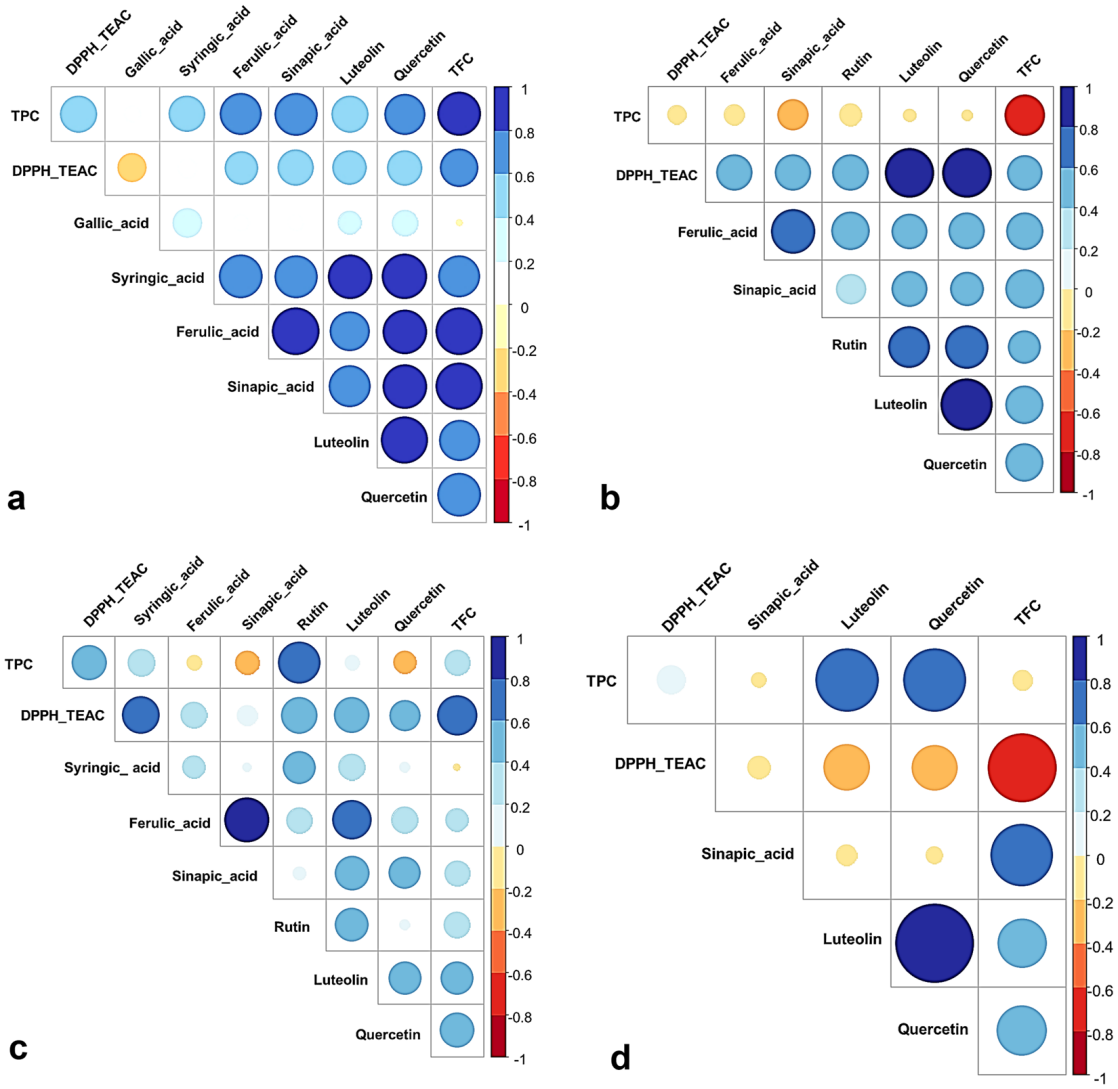
Pearson’s correlation between active compounds and their antioxidant capacity in different plant organ extracts: (**a**) correlation in roots, (**b**) correlation in stems, (**c**) correlation in leaves, and (**d**) correlation in seeds. **TPC**: Total Phenolic Contents, **TFC**: Total Flavonoids Contents, **DPPH_TEAC**: antioxidant capacity. The intensity of the colour and the size of the circle are proportional to the correlation coefficients. To the right of the correlogram, the colour legend shows the correlation coefficients and the corresponding colours. The correlation was visualised by the ggcorrplot package.

The various plant organ extracts were tested for antioxidant ability using an *in vitro* antioxidant assay (DPPH). To estimate this antioxidant capacity, we calculated the percentage of inhibition of the free radical DPPH (I %) and the Trolox equivalent antioxidant capacity (TEAC). The Kruskal-Wallis test and ANOVA revealed a highly significant difference in TEAC among the *M. minima* organs (Supplementary Table S1). Leaf and seed extracts displayed the highest TEAC (Fig. 1c). For each tissue, ANOVA coupled with post hoc Tukey tests highlighted a significant difference in the TEAC between *M. minima* populations (p < 0.001; Fig. 3c, Supplementary Table S2). In the root extracts, populations from the lower semi-arid region (pop5, pop6 and pop7) presented the highest TEAC, whereas, populations from the higher arid region exhibited the lowest levels (Fig. 3c, Supplementary Table S3). In summary, pop5, pop6 and pop7 recorded a notable level of TEAC for all plant parts. Moreover, the association between TEAC and phenolic compound contents was performed by the Pearson correlation (Fig. 7). In the roots, Pearson correlation analysis illustrated a moderate positive correlation (+0.6) between TEAC and several phenolic parameters, including TPC, TFC, quercetin and sinapic acid (Fig. 7a). Further, in the stems there was a strong positive correlation (+0.9) between TEAC and luteolin and quercetin (Fig. 7b). In the leaves, there was a moderate positive correlation between TEAC and TFC (+0.7), syringic acid (+0.6), luteolin (+0.6) and TPC (+0.5; Fig. 7c). Unlike leaves, the TEAC in the seeds did not have any significant positive associations with phenolic compounds. However, there was a strong negative correlation (−0.9) between TEAC and TFC in the seeds (Fig. 7d).

### Discrimination of *M. minima* populations

We performed principal component analysis (PCA) using each compounds content that presented a significant difference between the studied populations (Supplementary Table S2) as a marker. Note that we did not include altitude and climate parameters in this analysis (illustrative factors).

#### Root data set

**Figure 8:**
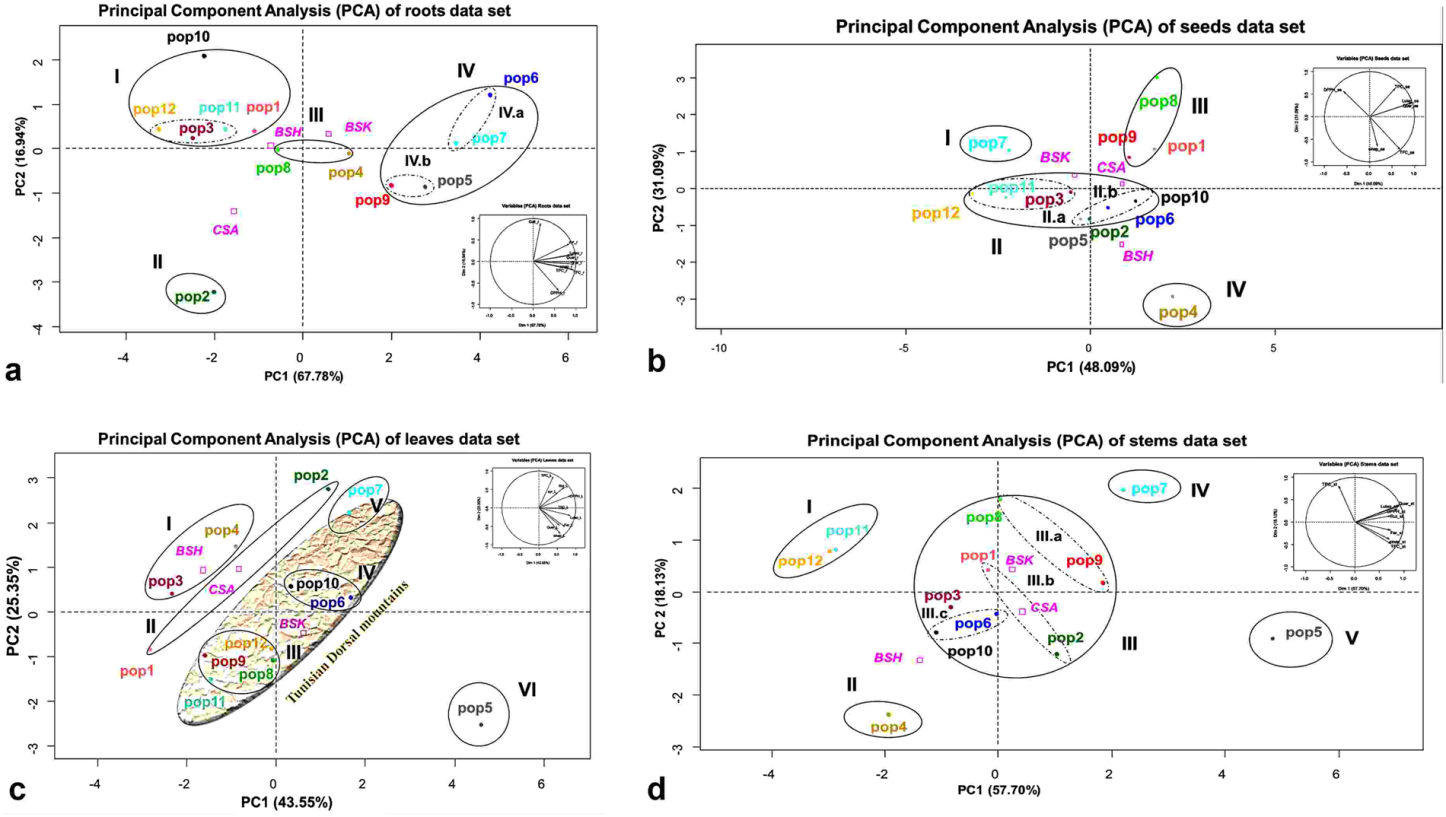
Principal component analysis of twelve *Medicago minima* populations based on their phenolic profiles in each plant organ. Each population is represented by a specific colour. (**a**) The principal component analysis differentiates populations based on their of roots contents (n = 12). The first three axes of PCA (PC1, PC2, and PC3) express 90.77% of the total variance. Therefore, four groups are distinguished (I, II, III, and IV (IV.a and IV.b)) along the first component gradient (PC1), which accounted for 67.78% of the variance. The second axis (PC2) represents 16.94% of variability. (**b**) The principal component analysis discriminates populations based on their of stems contents (n = 12). The variance accumulated by the three first PC is 86.06%. The PC1 participate by 57.7% of the total accumulated variance and discriminate populations in four groups (I, II (II.a and II.b), III, and IV) according to their TEAC, TFC, ferulic acid, sinapic acid, rutin, luteolin, and quercetin contents in the stems. The second component (PC2), with 18.13% of the variability, differentiates populations according to their TPC. (**c**) The principal component analysis separates populations in six groups (I, II, III, IV, V, VI) according to their leaves contents (n = 12). The three axes represent 82.30% of the total variance. The PC1 participates with 43.55% of the total accumulated variance and discriminates populations according to their TEAC, TFC, ferulic acid, sinapic acid, luteolin, rutin, and quercetin contents. The second axis (PC2) with 25.35% of the variability differentiates populations according to their TPC. (**d**) The principal component analysis of seeds dataset discriminates populations into five groups (I, II, III (III.a, III.b, and III.c), IV, and V) (n = 12). The principal component analysis of seeds dataset represents 95.24% of the total variance and discriminate four groups. The first axis (PC1), absorbs 48.09% of variance, and differentiates populations according to their TFC, luteolin and quercetin contents. The second axis (PC2) with 31.09% of the variability separates populations according to their TPC and TEAC contents. Figures were produced by R program packages (Rcmdr, car, and RcmdrMisc).

The use of the root data as quantitative variables in PCA analysis discriminated populations according to their root phenolic profiles (Fig. 8a). Populations from the arid region (pop3, pop12 and pop11) and the semi-arid zone (pop10 and pop1) constructed group I (Supplementary Table S3). Those populations exhibited the lowest phenolic compound contents and antioxidant capacity in roots but substantial gallic acid content (Figs. 3, 6, 8a). Group II included pop2, which had the lowest phenolic compounds in roots. The populations nearest to the centre of the PCA (pop8 and pop4) constructed group III, also called the null group. Populations from the semi-arid region (pop6, pop7, pop5 and pop9), all of which had relatively high phenolic contents, constituted group IV. According to the second axis (PC2), group IV was divided into two subgroups. On the higher side, pop6 and pop7 had higher contents of all phenolic compounds and were established as subgroup IV.a. On the opposite side, subgroup IV.b included pop9 and pop5, both of which had a low gallic acid content.

#### Stem data set

The stem data used in PCA classified populations into five groups. Group I represented pop11 and pop12 and was correlated negatively to first component (PC1) and positively to the second component (PC2) (Figs. 3, 6, 8b). These populations presented a relatively high TPC; they are from the arid region with a cold-steppe climate. Group II contrasted with the first along the second axis (PC2); it included pop4 from the arid zone with a hot-steppe climate (Supplementary Table S3). Therefore, the nearest populations to the centre of PCA constructed group III, which was subdivided into three subgroups (Fig. 8b). The first subgroup (III.a) was positively associated with all principal components and included pop8 and pop9, both of which are from the higher altitude of the semi-arid area with a cold-steppe climate. The populations from the low-altitude region with a Mediterranean climate constructed the second subgroup (III.b). Those populations came from two provenances, pop1 from the middle-semi-arid region with higher TPC and pop2 from a lower-semi-arid area (Fig. 3, Supplementary Table S3). Group IV contrasted with group II; it comprised pop7, which exhibited the highest levels of stem phenolic compound contents (Fig. 8b). Similarly, pop5, which originated from the lower semi-arid area with a cold-steppe climate, represented group V; it contrasted with group I (Fig. 8b, Supplementary Table S3).

#### Leaf data set

The leaf data set generated six groups in the PCA. Pop3 and pop4 from the arid region with a hot-steppe climate formed the group I (Fig. 8c, Supplementary Table S3). Those populations presented high TPC but lower contents for the other identified compounds (Figs. 3, 8c). Two contrasted populations (pop1 and pop2) that belong to the semi-arid with Mediterranean climate formed group II. Populations from the arid region with a cold-steppe climate (pop11 and pop12) and from lower-semi-arid region with cold-steppe climate (pop8 and pop9) represented group III (Fig. 8c, Supplementary Table S3). These populations exhibited the lowest leaf phenolic compound contents (Figs. 3, 8c). Group IV was represented by pop6 and pop10; these plants had the highest contents for all measured compounds and belong to the lower-semi-arid region with a cold-steppe climate. Group V was represented by pop7 from the lower-semi-arid zone with a Mediterranean climate; it had the same phenolic characteristic as group IV (Fig. 8c, Supplementary Table S3). Groups III, IV and V represented the Tunisian dorsal mountains (Figs. 2, 8c). Notably, group VI (pop5) contrasted with group I.

#### Seed data set

PCA of the seed data set differentiated four groups. Group I, represented by pop7, was characterised by a low TFC and a high TPC content (Figs. 3, 8c). Group II is in the middle of the PCA; it was formed by two subgroups. Subgroup II.a represented populations with low phenolic compound contents (pop11, pop12 and pop3), all of which belong to the higher arid region (Figs. 3, 6, 8d). Subgroup II.b contained populations from the low-semi-arid regions (pop2, pop5, pop6 and pop10) with a moderate TFC. Group III represented populations with a high content of all studied phenolic compounds (pop9, pop8 and pop1). Finally, group IV contrasted with group I; it was represented by pop4 from a higher-arid region with an arid-hot-steppe climate (Fig. 8d, Supplementary Table S3).

#### All organs data set

PCA analysis of the studied populations distinguished four groups. Group I represented pop9, pop6, pop7, pop8 and pop5, all of which contained the highest phenolic compounds contents and had a good antioxidant capacity (Figs. 3, 6). This group resides in the lower-semi-arid region and belongs to the BSK climate with a high altitude. Group II included pop11 and pop12 from the higher-arid region with a BSK climate; they exhibited relatively high TPC in stems. Groups III and IV represented the remaining populations; they were opposite to group I and characterised by low phenolic compounds contents. Group III was near to the centre of the PCA (Supplementary Fig. S1); these plants belong to the temperate-dry-hot-summer (CSA) with a low altitude (100–320 m). In contrast, group IV belongs to the hot-steppe climate (BSH) with the lowest altitude (<200 m). These findings indicate that PCA separated the different populations into four groups according to their active compound contents, which was consistent with their provenance environment, climate and altitude.

## Discussion

During the current study, secondary metabolites were examined in *M. minima* species in order to highlight their phenolic potential and identify some of their phenolic compounds already acquired as a basis for medicine or food and feed. We established the phenolic composition of four organs in 12 Tunisian *M. minima* populations from arid and semi-arid regions and evaluated their antioxidant activity.

### The distribution of phenolic compounds in plant organs and their antioxidant capacity

The obtained results indicate that *M. minima* roots, stems, leaves and seeds highlighted various levels of TPC, TFC and total antioxidant capacity. This variability has been also reported in *Medicago sativa* and *Cynara cardunculus* L.^26,27^. Focusing on two *Medicago* species, *M. minima* presents a higher TPC compared to *M. sativa*, which has obtained great advantages in agro-industry^24^. However, the highest phenolic content organ in *M. minima* was the leaves, whereas the seeds are the richest organ in *M. sativa*^26^. Like many other species (*C. cardunculus* L. and *M. sativa*), *M. minima* seeds had the highest total flavonoid content compared to all plant parts^26,28^. However, in *M. minima*, the leaves contained more TFC compared to the roots and stems, unlike *M. sativa*^26^. Hence, there is a difference in the distribution of the phenolic compounds in the plants’ organs of neighbouring and non-neighbouring species^24–28^. Furthermore, the large variability in TFC in the roots may reflect the interaction between *M. minima* plants and their environment (soil). As signal molecules, flavonoids play a major role in promoting nodular formation in roots cell by symbiotic bacteria commonly known as rhizobia^29,30^. Flavonoids are widely distributed in all legume tribes. They intervene in the environment-plant connection, including antimicrobial, anti-herbivore and antioxidant effects^31^. Therefore, the fact that the highest antioxidant capacity has been found in *M. minima* seeds and leaves suggests that the accumulation of flavonoid in the aerial organs of *M. minima* constitute a part of their defence strategy.

As we previously mentioned, *M. minima* plants accumulate phenolic and flavonoid compounds in their leaves and seeds more than in other organs. Therefore, investigating each pant organ’s phenolic composition allowed us to clearly understand this variation. Our HPLC analysis identified 70% of inspected phenolic compounds. The HPLC profiles confirmed that roots and seeds constitute the richest organs. Major compounds, such as gallic acid, luteoline and quercetin, were present in *M. minima* plants’ organs, with an average concentration above 250 µg/g DM. Nevertheless, the minor compounds, namely ferulic acid, rutin, sinapic acid and syringic acid, had an average concentration less than 300 µg/g DM. Such phenolic compounds have been the target of considerable interest in the agro-industry^17^, as well as in medicine^15,18,19^. They can also be used as antimicrobial additives and antioxidants in the food industry, especially gallic acid, which is considered to be good at inducing sweetness^17^. Almost all of the major and minor compounds are available in the *M. minima* roots, except rutin. All compounds showed a large variation within *M. minima* roots. Those compounds are immensely important because they have various functions in symbiosis (plant-microbe interactions), which influences the rhizobium growth^29,30^. The phenolic composition variability among plant parts is highlighted by the presence of gallic acid only in the roots, and syringic acid only in roots and leaves extracts. In soybean nodules, gallic acid reportedly acts as the primary antioxidant compound^30^. Nevertheless, in the root exudates of holm oak (*Quercus ilex*), the metabolism of gallic acid is activated and deactivated in response to drought stress^32^. Our results are consistent with this previous observation, which applied drought stress. The variability among plant parts was highlighted by the presence of the rutin only in the aerial organs of plants. *M. minima* stems and leaves exhibited the same identified phenolic compounds^33^ except syringic acid; this discrepancy constitutes the difference between these two aerial organs. Those compounds (rutin and syringic acid) were also identified in the aerial organs of *Medicago truncatula*^33^. Moreover, *M. minima* leaf extracts contained rutin and syringic acid, both of which are absent in *M. sativa* leaves extracts^26,34^. Rutin comprises one of the important constituents of apples and has multiple pharmacological activities with a wide array of biological activities^35^. Similarly, syringic acid has been described as a potential pharmaceutical compound that has the ability to modulate enzyme activity, protein dynamics and various transcription factors that are involved in diabetes, inflammation, angiogenesis and cancer^22^. Our results underscore the difference among the *Medicago* genus and highlight the importance of *M. minima*, especially in foods and other interest fields^21,22,24,25,35,36^. Furthermore, the presence of the ferulic acid in the aerial parts of the plant and roots demonstrates the potential of this plant in medicine^37^ and the cosmetics industry^38^. Due to the presence of syringic acid and ferulic acid in *M. minima* leaves, this unused plant can be an excellent source of therapeutic and cosmetics compounds that can prevent skin carcinogenesis^21,36–38^. Besides, luteolin, quercetin and sinapic acid are present in all *M. minima* plant parts. The unavailability of the ferulic acid in the *M. minima* seeds is common with black beans (*Vigna cylindrical*), whereas it is present in other legume species such as soybean (*Glycine max*), peanuts (*Arachis hypogaea*) and mung beans (*Vigna radiata*)^39^. However, sinapic acid has the potential to exert various health benefits^20^. This phenolic acid was identified in *M. minima* seeds (69.92 µg/g DM), and previous studies have reported it in other legume seeds as peanuts, mung beans and soybean^39^. However, this compound does not apparently contribute to antioxidant activity in several important consumable plants’ organs or foods, such as honey and *Lathyrus cicera* seeds^40,41^. This observation situates *M. minima* in a very interesting position for its exploitation as a plant with specific biological activities. In addition, luteolin and quercetin present in *M. minima* seeds have also been identified in *L. cicera* seeds^32^.

Some studies have declared that the antioxidant activity of such plant organs can be correlated with the presence of such phenolic compounds, mostly because the high value of TPC and TFC indicates high DPPH, ABTS and FRAP activities^42^. Our results suggest the presence of a significant positive correlation between antioxidant capacity in *M. minima* leaves and roots and their phenolic and flavonoid contents. The same results have been reported in nut extracts^16^, tealeaf extracts^42^ and berry extracts of *Hippophae rhamnoides*^43^. However, there was no correlation between *M. minima* seed extracts and their high antioxidant capacity, similar to findings for other plant extracts^44–46^. Some studies have reported that the correlation between phenolic compounds and antioxidant potential is not conclusive^24,26,27^. This eventuality suggest that this antioxidant activity is generated by an unidentified compound. Therefore, phytochemical compounds can be the key of the antioxidant elements in *M. minima* seeds^46^. Indeed, antioxidants play an important role in neutralising the harmful effect of oxidative stress and preventing various diseases, such as obesity, cardiovascular and neurodegenerative diseases^18,20,22^. Due to the presence of those compounds in the various *M. minima* organs, this plant should be useful and beneficial to human health^5,19,26,36^. The phenolic profiles in *M. minima* plant organs illuminate the exploitation potential of this unused plant^21,22,35–38^ and reflect the relationship of this plant with its ecosystem^29–32^.

### The effects of the microenvironment variation on the phenolic composition in four plant organs

Water availability, temperature, altitude, UV, soil and humidity constitute important factors that affect metabolism and secondary metabolite accumulation^4,11,33^. Thus, as a survival strategy, environmental variations in different provenances lead to the variation of the phenolic compounds of plants^4,33^. We distinguished the twelve populations selected from various climate environments in Tunisia with PCA based on phenolic profiles. Consistent with other studies, our finding suggests that the variation of the TPC and TFC among populations is not associated with geographic distance, but it can be related to their natural environments^11,47^. Therefore, it is important to note that in different plant organs there may be differences in the phenolic response to environmental stress^47,48^.

In roots, populations from the semi-arid region with a BSK climate presented the highest phenolic compound contents, except gallic acid. Populations from the arid region with a BSK climate had the highest gallic acid contents and the lowest levels of all other identified phenolic compounds. In this case, phenolic compounds seem to increase in populations adapted to the semi-arid regions. However, recent studies have shown that secondary metabolites (phenolic compounds) of *Quercus ilex* root exudate increase under stress conditions^32^. Hence, *M. minima* roots adapted to the semi-arid lands were under natural stress condition compared to those from the arid region. As an underground organ, the water availability, soil composition and microorganismal milieu can affect metabolism in the roots^11^. In addition, biotic stresses such as mycorrhisation raise the flavonoid levels in red clover roots^49^. Therefore, *M. minima* is a legume species that can make symbiosis connection (nodule) between their root and the soil rhizobium^29,31,39^. In addition, the cycle of nodule formation includes the production of flavonoids, betaines and phenolic acids by the plant as a signal to the microbial symbiont in root exudates^29,30,50^. Our finding suggests that the root phenolic contents are augmented in plants adapted to the lower-semi-arid region compared to those from the arid region. The availability of the water in the semi-arid region could therefore influence the oxygen availability, the rhizobium strain and the soil composition^30,50^. Thus, the roots adapted to the arid land conditions had the highest gallic acid roots contents, a phenomenon that might reflect the interaction between roots and soil rhizobium. Therefore, in drought conditions, the roots of *M. minima* could make more symbiotic connections by using gallic acid^29^. In addition, a study has shown that the root phenolic acid content decreased significantly due to low temperatures^51^, which concord with our results founded by a natural adaptation process. Moreover, Gargallo-Garriga *et al*. have concluded that even small variations in water availability can have a measurable influence on the composition of root exudates^32^, which we showed in our study. For example, pop4, which was collected near of a water dam, accumulated more phenolic compounds compared to those adapted to same microenvironment (arid region, low altitude, and BSH climate).

Concerning the stem, populations adapted to the semi-arid region with a BSK climate had the highest contents of identified compounds and the lowest TPC. In contrast, populations located in the arid region with a BSK climate had the highest TPC and the lowest identified phenolic compound contents. Therefore, like *Gossypium hirsutum* and the peanut, *M. minima* stems from the arid regions increase their TPC as an adaptation form to the arid environment^13,52^. However, *M. minima* stems adapted to the semi-arid area had higher quercetin and luteolin contents compared with those from the arid zone. This adaptation process seems to be the result of the temperature rise response like that found in *Lactuca sativa*^11^.

In addition, the leaf phenolic composition varied depending on the adaptation to the environments, especially with the altitude. For example, some studies have shown that phenolic compounds, such as anthocyanins, increase when plants are exposed to UV-B radiation^53,54^. Indeed, ultraviolet (UV) radiation and atmospheric pressure increase with altitude^55^. Therefore, as a form of adaptation, higher-altitude populations (more exposed to UV radiation) could have more phenolic contents compared to lower-altitude populations^56^. In addition, according to our previous results, the original population from semi-arid environments could be considered naturally adapted to environmental stress. Consequently, under these two conditions (UV radiation and water availability), only *M. minima* individuals that have the highest phenolic compounds in their organs could survive^11,53,54^. Multiple combined environmental stresses can exert different impacts on the phenolic composition of the exposed plant organs^11,54^. Therefore, in response to the combined environmental conditions, the leaf TPC discriminated the studied *M. minima* populations according to their phenolic profiles. The semi-arid region populations exhibited the highest TPC compared to the arid region populations. These two contrasted groups occupy a higher altitude with a BSK climate and construct the Tunisian dorsal mountains.

The populations were not well-discriminated based on seed data; they were entirely based in the centre of PCA. Therefore, the TPC did not contribute to this differentiation. Usually, TFC could differentiates populations of the arid land from those of the semi-arid. Based on the literature, flavonoids are present in most plant seeds as secondary metabolites^41,47^. These compounds defend the seeds from pathogens and predators and participate in seed maturation and dormancy^57^. Flavonoids certainly have an important role in seed germination; hence, we found them in many seeds, especially in the *Medicago* genus. Indeed, seeds of this species can cope with possible infecting because of their long dormancy cycle before germination^57,58^. Flavonoids also play an important role in seed germination^57^. Our results illustrate that the seeds from semi-arid regions contained the highest TFC. This finding might provide an idea of the seed germination rate. Moreover, MacGregor *et al*. has demonstrated that during seed maturation, low temperatures cause an increase in gene expression of enzymes involved in flavonoid and procyanidin biosynthetic pathway^59^. As a result, *M. minima* plants adapted to the semi-arid regions accumulate flavonoid compounds in their seeds more than plants from the arid region.

The integration of all data organs in one PCA allowed us to understand the *M. minima* adaptation strategy to their different natural environments. Our finding indicates that the highest phenolic compounds contents are observed in group I, which includes populations from the semi-arid area with BSK climate and altitude higher than 550 m. Similar results have been reported in *Potentilla fruticosa*^56^. Indeed, these populations have the highest levels of all compounds participating to the construction of PCA. According to the PCA, the most abundant *M. minima* populations in terms of active compounds reside in a BSK climate with a higher altitude (groups I and II). In this case, the higher altitude with a BSK climate could promotes the persistence only of *M. minima* plants with the highest seed TFC, stem TPC and leaf quercetin content^11,53,56,59^. The discrimination of the first and the second group highlighted the difference between the two microenvironments (arid and semi-arid regions) from the same climate (BSK). Populations from the arid region formed group II that was characterised by the lowest levels of root TFC and TPC, stem TFC and some identified compounds. Populations from the low altitude with a CSA or BSH climate constructed groups III and IV, respectively. Accordingly, warming (temperature increase) decreases phenolic compounds and increases volatile terpenoids in plants^11,12^, but these changes are not concordant with our observation of the adaptation process of *M. minima* plants. Therefore, root phenolic profiles could be the major parameters that differentiate populations from arid and semi-arid lands, while the stems could be the parameters that differentiate BSK climate populations from CSA and BSH climate population. On the other hand, the phenolic compound content decreases with altitude adaptation.

As many studies have reported, environmental factors influence secondary metabolism and antioxidant activity^12,56^. Our results correspond to these previous studies. Specifically, we found that plants adapted to a BSK climate accumulate more phenolic compounds compared to those from the BSH and CSA. Thus, the phenolic composition of each plant organ varies with climate adaptation. Like many other studies, ours results underscore the important heterogeneity of genetic resources, environmental adaptation and geographical location in the distribution of the phenolic compounds in plants^46,47,60^. Therefore, there is strong evidence that the variation in the secondary metabolite production and composition, and their potential activity among *M. minima* populations, could be due to an environmental adaptation process^56,60,61^. Based on our results, phenolic compound contents were successfully used as biochemical markers to discriminate natural *M. minima* populations. Moreover, the distribution of these compounds in different plant parts could provides an alternative for selection and engineering programmes for the valorisation of this unused plant in the interest fields.

## Conclusions

In conclusion, the present work is the first comprehensive study performed on the phytochemicals and antioxidant capacity of *M. minima* organs. The TPC, TFC and antioxidant capacity measured in *M. minima* roots, stems, leaves and seeds highlight their phenolic potential. We determined a linear relationship between TPC and DPPH antiradical capacity. Hence, the identification and the quantification of four phenolic acids and three flavonoid compounds in four various organs could open myriad potentials in exploitation of this unused plant. In addition, the impact of the microenvironment variation on the plants’ adaptation and their phenolic composition in four organs have been rarely studied. We showed that even a small difference in the availability of water in the plants’ growth region could strengthen the presence of some plants with a particular root phenolic composition. In addition, we found that the phenolic composition of the aerial plant organs (leaves and stem) differs among populations that originate from various altitudes. Therefore, the leaf phenolic composition could be a discriminative factor of the geographic origin (Tunisian dorsal mountains), the climate (BSK climate) and the altitude. Overall, populations with considerable phenolic composition are adapted to a BSK climate (arid-cold-steppe) with a higher altitude. The phenolic profile of the studied four organs opens a broad perspective to discover new phenolic potential.

Focusing on the *M. minima*, it has emerged as a species that is rich in high polar compounds with free radical scavenging properties. However, *M. minima* has not attracted the attention of the agricultural and agronomy industries. Therefore, it deserves special attention for their phytochemical potential. Our findings can provide useful information for the selection programme and exploitation and valorisation of these potential compounds in interesting fields for development sustainability.

## Material and Methods

### Reagents and solutions

Methanol (MeOH) Multisolvent HPLC grade purchased from Scharlau was used for the extraction of phenolic compounds. Folin-Ciocalteu reagents and analytical grade gallic acid as the standard were purchased from Sigma-Aldrich. DPPH (2,2-diphenyl-1-picrylhydrazyl) and Trolox (6-hydroxy-2,5,7,8-tetramethylchromane-2-carboxylic acid) powder was purchased from Sigma-Aldrich. Gallic acid, gentisic acid, caffeic acid, syringic acid, ferulic acid, sinapic acid, rutin, quercetin, luteolin and kaempferol that were used as standards were also purchased from Sigma-Aldrich.

### Plant material

The *M. minima* seeds used in this study were collected during the plant’s growing season in June. They were subsequently conserved in a cold storage room until use. The 12 studied populations were selected from dispersed sites through arid and semi-arid areas and covering regions on both sides of the Tunisian dorsal mountains (Siliana, Kairouan, Sousse and Kasserine; Fig. 2). The geographic distances between seeds are summarised in a matrix table (Supplementary Table S4). The collection site details are described in Supplementary Table S3.

Twenty seeds per population were sprouted in a petri dish at room temperature. After one week, only 10 plants were transplanted into pots filled with soil and peat (2:1). With regular irrigated, all plants were grown in a greenhouse for 3 months under uniform conditions (24/18 °C, 16/8 h photoperiod, light/dark). The collected plants of each population were cleaned in distilled water to remove the soil. Plants representative of each population were sorted into organs (stems, leaves, roots, and seeds) and pooled. The separated samples were then freeze dried. The various dried samples were stored and protected from light and moisture for further analysis.

### Phenolic extraction

Several studies have aimed to develop extraction systems (methods and solvent extractor) that provide the optimal parameters to obtain a large quantity of active compounds^1,26,62,63^. As many studies have reported, alcoholic solvent produces the richest flavonoid content in some *Medicago* species, while the hydro-alcoholic show the highest content in *M. minima*^1,62^. Polyphenols are relatively polar compounds and are easily soluble in this solvent system. Further, this safe solvent makes it possible to translate the extraction system to the industry^64^. As previously stated, the perfect solvent for *Medicago* species is hydroalcoholic with an alcohol proportion between 70 and 90%^1,62^. Therefore, our extraction process utilised methanol (80%) as a solvent. The extraction followed a previously described procedure^26^ with some modifications. The various dried samples were milled to powder, using liquid nitrogen and a mortar, and then stored in dark plastic tubes at room temperature in a desiccator. Total phenolic compounds were extracted by maceration of 0.2 g of dried plant material in and 80:20 (v:v) MeOH:water mixture for 16 h with stirring in the dark and at room temperate. After centrifugation of the extracts, the supernatant was filtered through a PTFE membrane (0.45 µm). Subsequently, we reduced all extracts to residue using a rotary evaporator (Heidolph HB digital) set under reduced pressure to 40 °C. We then calculated the ratio between the weight of dried extract and the initial plant weight. The residues from the dried crude extracts were suspended in a volume of methanol to prepare 20 mg/mL extract solutions in the solvent. We stored all extracts in the dark at 4 °C until analysis.

### Determination of dry matter (DM)

We dried the different plant parts in the oven at 105 °C until weight stabilisation. The difference in weight was used to determine the dry matter in 0.2 mg of sample.

### Determination of TPC

We used the Folin-Ciocalteu colourimetric method based on the reduction of a phosphowalframate-phosphomolybdate complex by phenolics compounds to blue reaction products^65^ to determine the TPC of leaves, stems, roots and seeds. Five hundred microliters of diluted sample extract (1/10) were added to 1 ml of Folin-Ciocalteu phenol reagent (1/10 diluted in deionised water). After an 8 min incubation in the dark, 1 ml of sodium carbonate solution (7.5%) was added to the mixture. After incubation for 30 min in the dark and at room temperature, we used slight centrifugation to eliminate the excess of Na_2_CO_3_ and measured the absorbance at Y = 765 nm. By using an analytical concentration grade of gallic acid (0–100 µg/ml) as a standard, we expressed the TPC for each sample as milligrams of gallic acid equivalents per gram of dry weight (mg GAE/g DW). We performed all analysis with triplicate experiments.

### Determination of TFC

We determined the TFC of all organs using a modified aluminium chloride colourimetric method^66^. We added 0.5 ml of each sample was added to 0.5 ml of 2% AlCl^3^ (solubilised in methanol) and measured the absorbance by spectrophotometry at 420 nm after 30 min. We used rutin for a calibration curve in order to determine the flavone content of each extract.

### Identification and quantification of some phenolic compounds by high-performance liquid chromatography method coupled with a diode array detection (HPLC-DAD)

In order to investigate the secondary metabolite composition of *M. minima* extracts, we used HPLC-DAD. The phenolic compounds standards included gallic acid, gentisic acid, caffeic acid, syringic acid, ferulic acid, sinapic acid, rutin, quercetin, luteolin and kaempferol. We utilised an Agilent 1260 infinity HPLC (Agilent Technologies Inc.) equipped with a model G4212B DAD, autosampler (G1329B), quaternary solvent pump (G1311B) and column compartment (G1316A). We separated *M. minima* extracts by the injection of 5 µl of the sample on a Kinetex 5 µm EVO C18 100 A column 150 × 3.0 mm (phenomenex). The solvents were 0.1% formic acid (A) and acetonitrile (B). These solvents flowed at 1 ml/min and the gradient was initiated by 95% (A) for 2 min, then decreased as follow: at 8 min 80% (A), at 11 min 74% (A), at 15 min 40% (A) and at 17 min 0% (A). We maintained the last condition for 2 min and then returned to the initial condition, 95% (A), within 4 min. We detected phenolic compounds from 220 to 640 nm by UV detectors. We used Agilent ChemStation software for data collection and analyses. We identified the standards based on their retention time and spectra range to generate the calibration curve. We used the calibration curve of standards that showed great linearity to quantify the corresponding compounds on *M. minima* extracts (Supplementary Table S5). Caffeic acid, gentisic acid and kaempferol were not identified in any extracts of different *M. minima* plant organs; only the identified compounds are listed in Supplementary Table S5.

### Antioxidant activity by free radical scavenging (DPPH)

To determine the free radical scavenging capacity, we used an *in vitro* antioxidant assay (with DPPH) according to Sanchez-Moreno *et al*.^67^. According to previous studies, the radical DPPH is stable in the alcoholic solvent at ordinary temperature with a very characteristic blue colour. However, in the presence of an antioxidant, it will be reduced and its colour will change. In this case, the antioxidant activity is measured with a spectrophotometer at 517 nm. In brief, we separately added 0.5 ml of diluted extracts samples, 0.5 ml of methanolic solution of TROLOX (0.1 mM) and 0.5 ml of MeOH (80%) to 0.5 ml of methanolic solution of DPPH (0. 2 mM). We vigorously vortexed the mixtures and incubated them in the dark for 30 min. We measured the absorbance at 517 nm against a blank (80% MeOH). We estimated the radical scavenging activity was estimated in terms of percentage of inhibition of the free radical DPPH (**43**) using the following equation:

$${\rm{I}}( \% )=[({\rm{A}}0-{\rm{Ac}})/{\rm{A}}0]\ast 100,$$

where A0 is the absorbance of the negative control of reaction (DPPH solution + MeOH), and Ac is the absorbance of the test samples.

With the intention of estimating the antioxidant contents of each extract, we determined the calibration curve from TROLOX solution by the plotting different TROLOX concentration against their corresponding percentage of inhibition of the free radical DPPH. The DPPH results are expressed as mg of Trolox equivalent antioxidant capacity (TEAC).

### Statistical analysis

We determined the analytical curves for TPC, TFC, antioxidant activity and HPLC-DAD (Supplementary Table S5) and quantified the corresponding compounds in all samples in order to estimate the contents in each sample. According to linear equations for each calibration curve of equivalence compounds, we established gallic acid (TPC), rutin (TFC) and standard (HPLC) contents. All data are reported as mean ± standard deviation of triplicate experiments. In order to compare the four organs and to prove that the differences between these organs are not an accident or due to a chance, we utilised the Kruskal-Wallis test. This test rejects the hypothesis that the organs are from identical populations. Moreover, we used ANOVA (at a significant level of p < 0.05) coupled with multiple comparisons of means (Tukey Contrasts) to investigate: the differences between different organ assays and to compare selected *M. minima* populations. In addition, we completed discriminative analysis, namely PCA, for each organ by the using TPC as a matrix. We evaluated the association between variables by the Pearson correlation method. The levels and the statistical analysis (ANOVA, Kruskal-Wallis test and Tukey test) are shown in Supplementary Tables S1, S2. We used R (version 3.5.1) for all statistical analyses. The utilised packages were: Agricolae, Rcmdr, car, RcmdrMisc, corrplot, tidyverse, hrbrthemes, ggplot2 and RColorBrewer^68–71^.

## Supplementary information

Supplementary Information.
