# Supplementary material for: Influence of climate variation on phenolic composition and antioxidant capacity of Medicago minima populations
Source: Sci Rep. 2020 May 19;10:8293. doi: 10.1038/s41598-020-65160-4 (PMC7237653; doi:10.1038/s41598-020-65160-4)
Supplement: Supplementary file 1 — Supplementary Information. [file 41598_2020_65160_MOESM1_ESM.pdf]

Influence of climate variation on phenolic composition and antioxidant capacity of *Medicago minima* populations

Kabtni Souhir [1], Sdouga Dorra [1], Bettaib Rebey Ines [2], Save Mattew [2], Trifi-Farah Neila [1], Fauconnier Marie-Laure [2], Marghali Sonia [1*]

[(1)] *Laboratory of Molecular Genetics, Immunology & Biotechnology, Faculty of Sciences of Tunis, University of Tunis El Manar, Tunisia*

[(2)] *General and organic chemistry-volatolomics, Gembloux Agro-Bio Tech, University of Liège*

[*] *Corresponding author: E-mail: soniamarghali@yahoo.fr; sonia.marghali@fst.utm.tn*

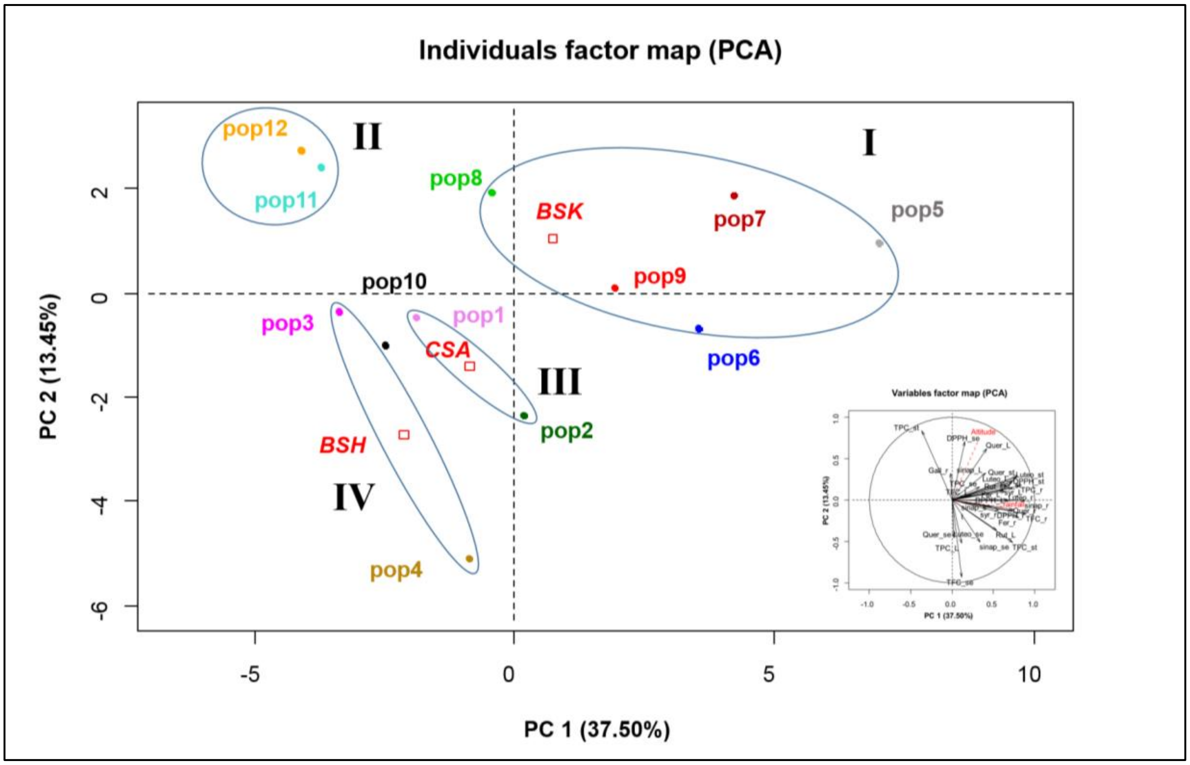

Supplementary Fig.S1 online. Principal component analysis of twelve *Medicago minima* populations based on their phenolic profiles in four plants organs. Each population is represented by a specific colour. The PCA analysis displays three principal components that represent 62.21% of the total variation and it highlights four different groups. Those groups are arranged along the gradient generated by the first component, which accounts for 37.50% of the total variance. Several phenolic compounds contents does not participate to the construction of the three PCA axes, such as the gallic acid in roots, all seeds data (except TFC and TEAC), and the TPC, TFC, sinapic acid contents in leaves.

Supplementary Table S1: *Kruskal-Wallis* and *ANOVA* analysis of phenolic compounds contents among *Medicago minima* organs (roots, stems, leaves, and seeds).

	Kruskal-Wallis test		ANOVA test			
	**chi-squared**	**p-value**	**Sum Sq**	**Mean Sq**	**F value**	**Pr(>F)**
**TPC**	16.397	0.000940	246.5	82.16	8.776	0.000113 ***
**TFC**	14.897	0.001907	218.3	72.76	5.287	0.00336 **
**TEAC**	22.056	0.0000634	34.67	11.557	13.09	0.00000304 ***
**ferulic acid**	34.077	0.000000190	6839	2279.7	7.553	0.000351 ***
**rutin**	44.152	0.00000000140	604059	201353	46.85	< 0.001 ***
**sinapic acid**	31.569	0.000000645	93102	31034	18.21	0.0000000793 ***
**syringic acid**	43.564	0.00000000186	683806	227935	18.96	0.0000000486 ***
**gallic_acid**	45.738	< 0.001	3713365	1237788	140.1	< 0.001 ***
**luteolin**	19.384	0.000227	2395554	798518	8.444	0.000152 ***
**quercetin**	13.357	0.00392	675354	225118	4.241	0.00102 *

***p-value<0.05, the degrees of freedom Df = 3*

Supplementary Table S2: ANOVA analysis of biochemical composition within *Medicago minima* roots, stems, leaves, and seeds extracts.

	**Roots**				**Stems**				**Leaves**				**Seeds**			
**Compounds**	**Sum Sq**	**Mean Sq**	**F value**	**Pr(>F)**	**Sum Sq**	**Mean Sq**	**F value**	**Pr(>F)**	**Sum Sq**	**Mean Sq**	**F value**	**Pr(>F)**	**Sum Sq**	**Mean Sq**	**F value**	**Pr(>F)**
**TPC**	441.1	40.1	1.37e+31	<0.001 ***	202.7	18.43	4.179e+31	<0.001 ***	426.5	38.77	2.969e+30	<0.001 ***	165.5	15.05	2.818e+30	<0.001 ***
**TFC**	1366	124.2	5.335e+30	<0.001 ***	81.2	7.381	1.179e+31	<0.001 ***	300.1	27.29	2.251e+30	<0.001 ***	69.46	6.314	6.178e+29	<0.001 ***
**DPPH**	35.69	3.244	1.51e+31	<0.001 ***	29.96	2.724	1.129e+31	<0.001 ***	41.36	3.76	1.874e+31	<0.001 ***	9.523	0.8658	2.811e+30	<0.001 ***
**I%**	8325	756.8	4.151e+30	<0.001 ***	8831	802.9	1.012e+31	<0.001 ***	8947	813.4	6.517e+30	<0.001 ***	22.01	2.001	1.799e+28	<0.001 ***
**ferulic acid**	34397	3127	5.327e+31	<0.001 ***	2370	215.5	8.942e+31	<0.001 ***	2918	265.2	2.413e+31	<0.001 ***	ND	ND	ND	ND
**rutin**	ND	ND	ND	ND	86118	7829	3.045e+31	<0.001 ***	481215	43747	1.326e+31	<0.001 ***	ND	ND	ND	ND
**sinapic acid**	227240	20658	3.602e+31	<0.001 ***	4457	405.2	5.146e+31	<0.001 ***	21648	1968	9.764e+31	<0.001 ***	5485	498.7	2.665e+30	<0.001 ***
**syringic acid**	227240	20658	3.602e+31	<0.001 ***	ND	ND	ND	ND	1528596	138963	4.858e+31	<0.001 ***	ND	ND	ND	ND
**gallic acid**	1165903	105991	3.901e+31	<0.001 ***	ND	ND	ND	ND	ND	ND	ND	ND	ND	ND	ND	ND
**luteolin**	11466634	1042421	4.343e+31	<0.001 ***	279726	25430	1.223e+31	<0.001 ***	603231	54839	1.923e+31	<0.001 ***	132675	12061	1.802e+30	<0.001 ***
**quercetin**	4010440	364585	4.81e+31	<0.001 ***	983533	89412	3.244e+31	<0.001 ***	1657861	150715	2.45e+31	<0.001 ***	355191	32290	2.272e+30	<0.001 ***

TPC *is expressed as mg.EAG/gDM,* ***TFC*** *is expressed as mg RE/gDM,* ***DPPH*** *is expressed as µg.TEAC/gDM, µg of standards/ ml,* ***DF*** *(degrees of freedom) =11,* ***ND:*** *not detected*, ***Signif. codes of p-value:*** *0 '***' 0.001 '**' 0.001 '*' 0.05 '.'*

Supplementary Table S3: Geographic origin and climatic data of twelve *Medicago minima* populations. ***According to Köppen-Geiger climate classification: CSA****: temperate dry hot summer,* ***BSH****: arid hot steppe,* ***BSK:*** *Arid cold steppe.*

Populations code/site	GPS	Locality	Geographic origin	Climate	Bioclimatic region	Altitude (m)
Pop1	36°24'14.7"N 9°14'42.8"E	Siliana	New-dogga	CSA	Middle semi-arid	320
Pop2	36°01'27.6"N 10°30'23.0"E	Sousse	Hergla	CSA	Lower semi-arid	100
Pop3	35°47'36.1"N 9°48'18.9"E	Kairouan	Ainjloula	BSH	Higher arid	190
Pop4	35°33'13.0"N 9°43'38.9"E	Kairouan	Barrage d'el haouareb	BSH	Higher arid	200
Pop5	35°49'38.7"N 9°37'01.8"E	Kairouan	Oueslatia	BSK	Lower semi-arid	550
Pop6	35°34'29.5"N 9°04'15.4"E	Kasserine	Jedelienne	BSK	Lower semi-arid	1,009
Pop7	35°51'52.0"N 9°12'36.4"E	Siliana	Makther	CSA	Middle semi-arid	870
Pop8	35°43'55.3"N 9°12'60.0"E	Siliana	Rouhia	BSK	Lower semi-arid	620
Pop9	35°34'49.1"N 8°41'50.4"E	Kasserine	Thela	BSK	Lower semi-arid	980
Pop10	35°15'52.7"N 8°45'27.4"E	Kasserine	Boulaaba-khamouda	BSK	Lower semi-arid	850
Pop11	35°01'34.5"N 8°39'07.8"E	Kasserine	Lac lafiel	BSK	Higher arid	720
Pop12	34°58'57.7"N 8°33'37.3"E	Kasserine	Thelept	BSK	Higher arid	920

Supplementary Table S4: Geographic distance among twelve *Medicago minima* populations. Distance in Km

Populations	pop2	pop3	pop4	pop5	pop6	pop7	pop8	pop9	pop10	pop11	pop12
**pop1**	119.4	84.5	104	71.8	93.2	59.8	74.2	103.6	133.8	162	169.5
	**pop2**	68	87	82.5	138	117.4	120.2	169.8	178.2	201.3	211.4
		**pop3**	27.5	17.5	70.8	54.4	53.6	103	111.7	135	144.6
			**pop4**	31.9	59.6	58.1	50.2	93.4	93.7	114	123.6
				**pop5**	56.5	36.8	37.7	87.5	99.5	124.8	134
					**pop6**	34.5	22	34	44.6	71.7	80.5
						**pop7**	14.7	56.2	78.4	106	114.6
							**pop8**	50	66.8	93.7	102.5
								**pop9**	35.3	61.7	67.6
									**pop10**	28.2	36
										**pop11**	9.54

Supplementary Table S5: Analytical curves for the different bioactive compounds

Tests	Compounds	Equation of the calibration curve	R^2
**TPC**	gallic acid	y= 0.00158x + 0.0289	0.9948
**TFC**	rutine	y= 0.00157x + 0.0088	0.9991
**DPPH**	Trolox	y=0.9588x	0.9451
**HPLC**	gallic acid	y= 7.8688x + 93.571	0.9618
	syringic acid	y= 5.6043x + 46.566	0.9915
	ferulic acid	y= 75.516x + 352.4	0.9922
	sinapic acid	y= 37.778x + 70.84	0.9853
	rutin	y= 18.675x – 36.702	0.9976
	luteolin	y= 55.424x – 32.354	0.9989
	quercetin	y= 41.629x – 18.852	0.9996

(n=3), ***TPC*** *is expressed as mg.EAG/gDM,* ***TFC*** *is expressed as mg RE/gDM,* ***DPPH*** *is expressed as µg.TEAC/gDM,* ***All HPLC standards*** *are expressed as µg of standards/ ml.*
